# Supplementary material for: Search as a simple take-the-best heuristic
Source: R Soc Open Sci. 2019 Oct 30;6(10):190529. doi: 10.1098/rsos.190529 (PMC6837208; doi:10.1098/rsos.190529)
Supplement: Supplementary materials for “Search as a simple take-the-best heuristic” [file rsos190529supp1.pdf]

Supplementary materials for *Search as a simple take-the-best heuristic*

Kyanoush Seyed Yahosseini and Mehdi Moussaïd

Center for Adaptive Rationality, Max Planck Institute for Human Development, Berlin, Germany

{yahosseini, moussaid}@mpib-berlin.mpg.de

September 4, 2019

Experimental paradigm

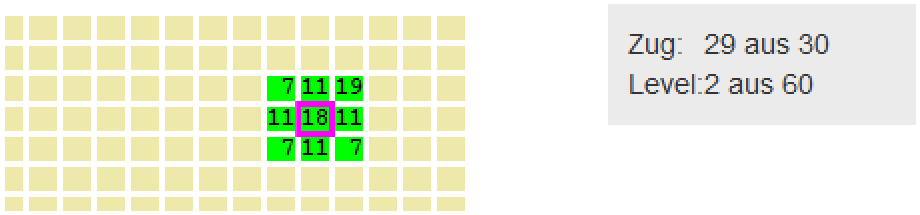

Figure A1: Experimental interface. The current position of the participant is indicated by the magenta square. The nine possible moving options and their respective payoffs are represented by the green cells. The grey text box on the right-hand side shows the number of remaining rounds (*Zug*) and landscape number (*Level*).

Patchy landscapes generation

In addition to the number of peaks in the landscape (i.e., rich vs. poor), we also varied the spatial distribution of the peaks to create patchy landscapes. Patchy landscapes are generated by the same procedure as the other

landscapes, with the exception that the peaks positions are sampled from a normal distribution with $mean = P$ and a standard deviation $sd = d$, where P is a random location in the landscape and $d = 6$ determines the peak's dispersion range.

Model comparison

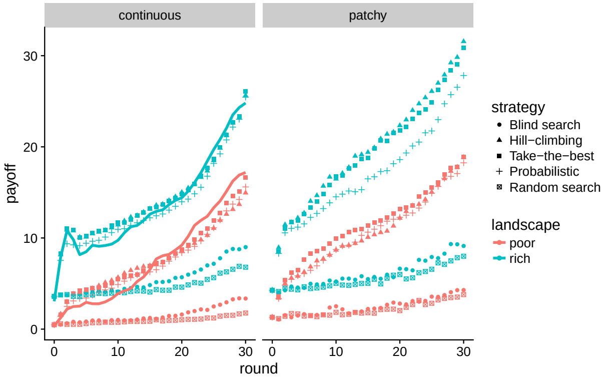

Figure A2: Payoff curves for different *exploration* mechanisms. Evolution of the average normalised payoff as a function of time as observed in the experimental data (plain line) and in simulations. Continuous landscapes (left) are those used in the experiment, whereas in patchy landscapes (right) peaks are clustered in one specific region of the landscape.

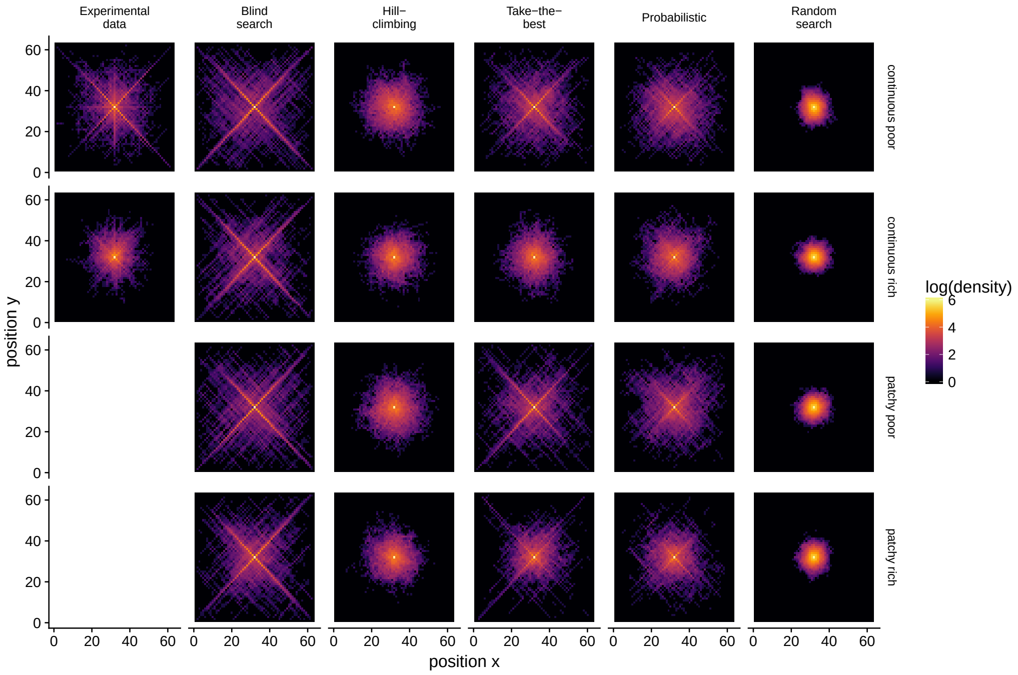

Figure A3: Density maps obtained for different *exploration* mechanisms (columns) and types of landscapes (rows) Experimental data are shown in the extreme left column. The colour coding indicates how often a given position (x,y) has been visited at the aggregate level, represented in logarithmic scale.

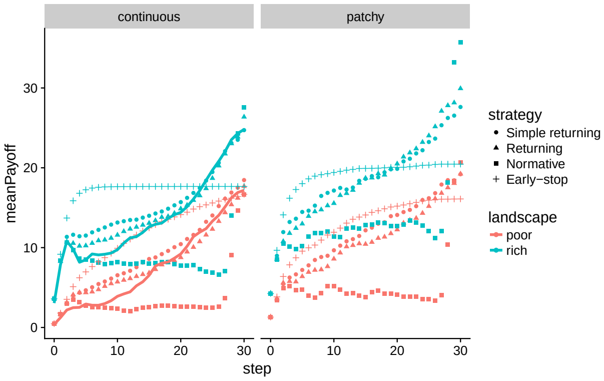

Figure A4: Payoff curves for different *exploitation* mechanisms. Evolution of the average normalised payoff as a function of time as observed in the experimental data (plain line) and in simulations. Continuous landscapes (left) are those used in the experiment, whereas in patchy landscapes (right) peaks are clustered in one location of the landscape.

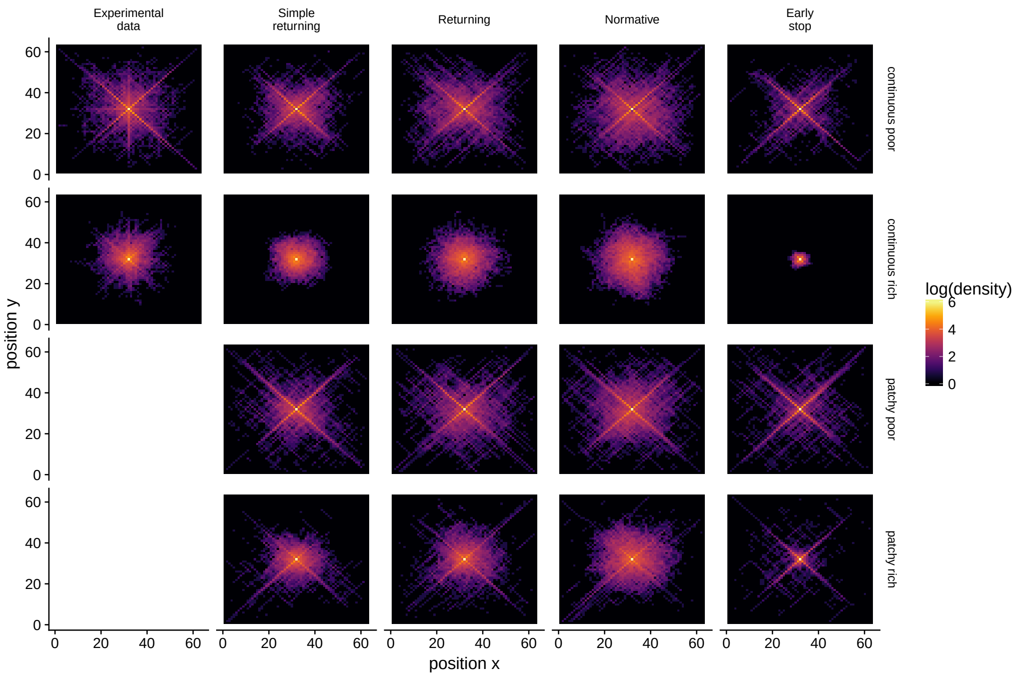

Figure A5: Density maps obtained for different *exploitation* mechanisms (column) and type of landscapes (row). Experimental data are shown in the extreme left column. The colour coding indicates how often a given position (x,y) has been visited at the aggregate level, represented in logarithmic scale.

Individual differences

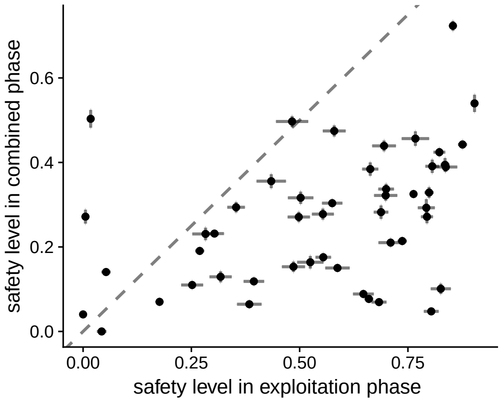

Figure A6: Safety levels displayed by the participants during the exploitation phase (x-axis) and the combined phase (y-axis). Each point indicates the average safety level of one participant. The error bars indicate the standard errors in the corresponding phase.

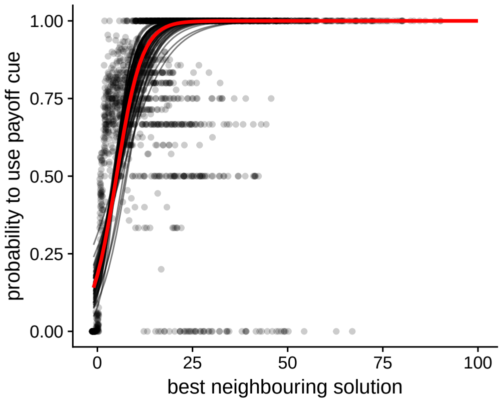

Figure A7: Relationship between the payoff of the most-rewarding neighbouring solution and the probability to rely on the payoff cue instead of the visibility cue. Each dot corresponds to one landscape. Each black line is the best fitted logistic function describing an individual's behaviour (mixed effect model with participants as a random effect, $BIC = 28949$). The red line indicates the best fitted logistic regression for the whole group, that is, not using the individual participant as a random effect ($BIC = 29261$).

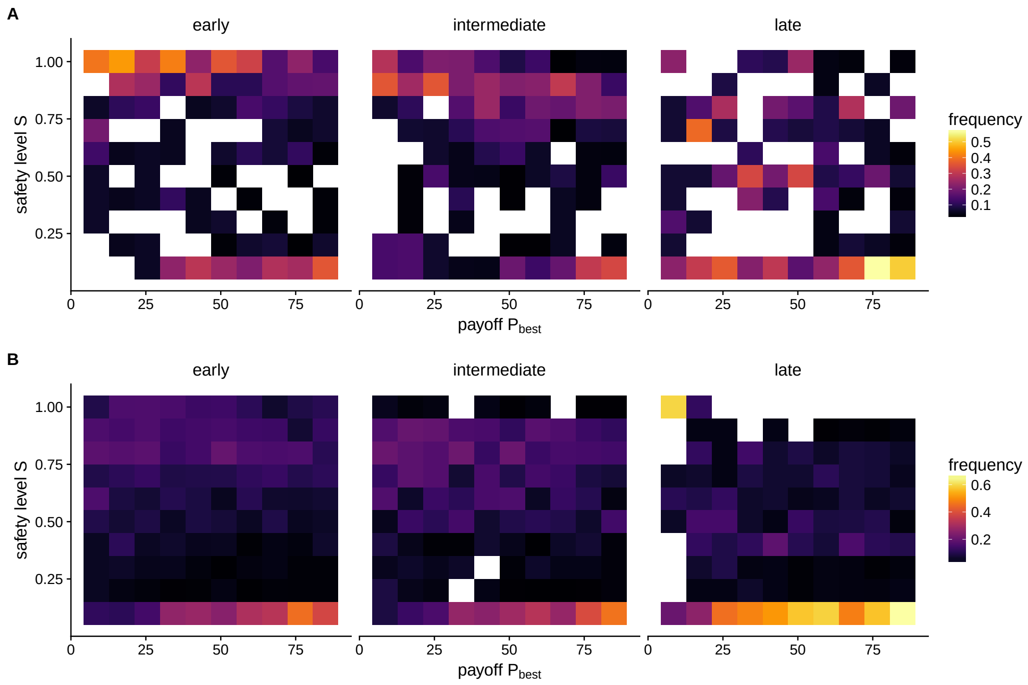

Figure A8: Safety level S as a function of the payoff P_{best} A) as observed in the experimental data, and B) as obtained from numerical simulations. Early, intermediate and late refer to the round where P_{best} is discovered, that is, round $1-10$, $11-20$, and $21-30$ respectively. The influence of remaining time on the safety level S is not captured by the numerical simulations.
